# Success, failure, and the imperative for justice in climate negotiations 

**DOI:** 10.2471/BLT.22.288351

**Published:** 2022-12-07

**Authors:** Laurie Laybourn-Langton

**Affiliations:** a Sustainability Accelerator, Chatham House, 10 St James’s Square, London SW1Y 4LE, England.

Recent United Nations Conferences of the Parties (COPs) have demonstrated that health professionals are playing an increasingly prominent role in calling for rapid action to address the climate crisis.^1^ COPs take place in the framework of the United Nations Framework Convention on Climate Change, the global treaty architecture for international climate negotiations and agreements. During the busy and crowded COP events, many interest groups voice their warnings and diplomatic demands.

The health community has made its voice heard at recent COPs. At COP26 in Glasgow, organizations representing over 46 million health-care professionals called for health and equity to be central to more ambitious action.^2^ More recently, before this year’s COP27 in Egypt, over 250 health journals from all world regions urged emergency action in a joint editorial.^3^ These initiatives are examples of coordinated interventions by health-care professionals that focus on securing greater ambition from countries to meet global climate goals. These goals – to limit average global heating below 2 °C above pre-industrial levels, and preferably no greater than 1.5 °C – are mandated by the 2015 Paris Agreement, an international treaty adopted under the Framework Convention on Climate Change at COP21 in Paris. Unlike other international sustainability goals and indicators, such as the sustainable development goals, the Paris Agreement is legally binding: it requires governments to set nationally determined contributions to collective global emissions reductions that place the world on a trajectory to meet temperature targets.

The Paris Agreement legally binds signatories over a process, not outcomes. Contributions are self-determined.^4^ The procedural requirements of bringing these contributions are joined by a principle of progressive ambition and the powerful normative expectations that come with it. This principle influences each stage of a five-year cycle: contributions must be presented and progress transparently reported, after which a stocktake is used to assess progress towards the goals. Countries are then required to update their commitments. The stocktake also assesses progress on adaptation to the impacts of climate change and on support to low-income countries. However, the Paris Agreement has no recourse to directly sanction governments for failing to increase ambition.

The combination of nationally determined contributions, the progressive ambition principle, transparency about reporting on contributions and the stocktake process create a solid mechanism. This mechanism intends to boost ambition on long-term goals to reduce greenhouse gas emissions as well as on building resilience to the inevitable effects of the climate crisis, and increase support to low-income countries. This mechanism also creates a powerful opportunity to establish norms and apply pressure on governments to increase ambition. While the stocktake process of the Paris Agreement only began in 2022, similar dynamics have been driven outside of formal structures.

For example, in October 2018, the Intergovernmental Panel on Climate Change released its highly influential report on the goal of 1.5 °C, revealing that emissions would have to fall by almost half over the 2020s and to net-zero by the mid-part of the century to protect that goal.^5^ The explosion of climate activism since 2018 – epitomized by striking school children and direct-action protestors – might be partly attributed to the powerful normative expectations this report created, as well as to the increasing stream of scientific evidence, media reporting and political concern.

Another example is the concept of the emissions gap, that is, the difference between the trajectory of reductions needed to meet the Paris goals and those committed by the sum of nationally determined contributions. For the past 13 years, before COP meetings, the UN Environment Programme has released an overview of the difference between where emissions are predicted to be in 2030 and where they should be to meet goals. The predicted gap before this year’s COP27 in Egypt puts the world on track for a 2.4–2.6 °C temperature rise by 2100 (if promises are met),^6^ which is an absolute failure. Yet this figure is also a relative success, because between COP21 and COP27, the projected temperature rise has been revised down from 5 °C.^7^

The actions of health professionals around COPs can be understood as responding to the formal processes of the Framework Convention on Climate Change and, most recently, the Paris Agreement, as well as the general dynamics created by normative expectations around the climate crisis. In all cases, the health community is well positioned to play a vital role in increasing the pressure on governments that are not suitably ambitious; health professionals who take on this advocacy role could be trusted, high profile and relatively powerful actors within domestic and international contexts.

Indeed, the great promise of health advocacy is that it could establish a new norm that the climate crisis is, fundamentally, about health. Doing so could bring the urgent risks into greater focus, grounding them in lived experience and existing government concerns over population health, and would help us better understand the significant co-benefits from emissions reductions. Improved air quality alone could realize health benefits that easily offset the global costs of emissions reductions.^8^

While this evidence is now well established, health does not yet seem to be a driving focus of climate norms or feature within formal agreements. For example, health is only mentioned once in the Paris Agreement, in a generic reference to obligations on the right to health. Such a gap is partly a consequence of the relatively recent uptick in climate-health advocacy having come too late to significantly influence the Agreement. This lateness underlines the need for the health community to increase its efforts to influence governments through presenting evidence on the climate threat to health as well as the options for a sustainability transition that maximizes health co-benefits. Many opportunities exist for more health professionals to do so, including through the growing efforts of the World Health Organization, national associations and civil society organizations such as the Global Climate and Health Alliance.

Many in the health community recognize the imperative of driving progress within health systems. The imperatives set by the Paris Agreement have spurred coalitions to organize deals that drive decarbonization in specific countries and sectors, something which is outside the Agreement’s mandate – but that it effectively encourages. At COP26, 14 countries committed to reaching net-zero emissions in their health-care systems,^9^ which is a sign of progress. However, it is important that other countries join them and that public and private sectors come together to develop plans for decarbonization across health supply chains and systems that span within and across countries.

In addition to the imperative to promise and deliver emissions reductions, two interrelated issues dominate COP meetings and are of particular concern to health: justice and adaptation. Power imbalances work to exacerbate the inherent injustice of the climate crisis. Wealthy nations have disproportionately contributed to the cumulative emissions causing the problem. They economically benefited from doing so, which might partly reduce their vulnerability to worsening impacts. Because of their economic status, higher-income countries are in a powerful position. This position has been used to break promises to less wealthy, more vulnerable nations that called for more support to help adapt to the impacts of a warming world and for explicit funds to compensate for the loss and damage already caused.

These demands have often been framed as moral concerns. Those who caused the problem have an obligation to help those who barely contributed and suffer the most, including by paying reparations for the accumulating costs of the lucrative carbon largesse of industrialized countries. As health professionals know, improved adaptation is also needed to protect global progress on development, poverty reduction and public health – a progress increasingly eroded by the consequences of rising temperatures.^10^ Yet another reason exists for supporting the most vulnerable: no country or community can be fully protected from the consequences of climate change. 

The climate crisis has direct impacts – extreme shocks, like severe storms, or slow-burn problems, like water depletion – that have knock-on consequences for public health, economic stability and political cohesion. These consequences are not isolated to one place. Instead, they are transmitted and amplified through globalized economic and social systems.^11^ The coronavirus disease 2019 (COVID-19) pandemic is an example of these cascading, systemic risks. Warnings are growing of climate-induced risks to food systems, migration and the subsequent rise of political extremism. These risks are yet to feature prominently enough in narratives of the climate crisis consequences or as a central concern of policy-making.^12^

The main implication of an emerging era of severe cascading risks is that, in a globalized world, no nation can be truly insulated from the growing instability brought by rising temperatures. As shown during the COVID-19 pandemic and the imperative to vaccinate all populations, we are globally as strong as our weakest member. Major increases in adaptation financing and loss and damage compensation are therefore practical imperatives, not just moral necessities.

In the extreme, the destabilizing effects of the climate crisis could divert energy from reducing emissions, creating a destructive feedback loop in which managing the symptoms of the climate crisis swamps action on its root causes. Surviving this feedback loop could be the Paris Agreement’s greatest challenge. The role of health will be critical because healthier societies are more resilient societies. However, will health systems be robust in a world that has warmed by more than 1.5 °C? Which co-benefits of climate action are most likely to ensure societies are resilient to accelerating climate shocks and can stay focused on emissions reductions? What are the limits to adaptation? Health professionals should be asking these questions now. 
